# The roles of language and executive function in Mandarin-speaking children’s theory of mind development

**DOI:** 10.3389/fpsyg.2024.1354207

**Published:** 2024-06-12

**Authors:** Honglan Li, Man-Tak Leung

**Affiliations:** ^1^School of Foreign Studies, Nanjing University of Science of Technology, Nanjing, China; ^2^Department of Chinese and Bilingual Studies, Hong Kong Polytechnic University, Kowloon, Hong Kong SAR, China

**Keywords:** language, executive function, theory of mind, first-order false belief reasoning, second-order false belief reasoning

## Abstract

**Introduction:**

Research has indicated that language and executive function relate closely to first-order false belief reasoning, yet their roles in second-order false belief reasoning are under-explored, and their interplay in theory of mind development remains obscure.

**Methods:**

This study assessed 160 Mandarin-speaking preschoolers’ and early primary schoolers’ language, executive function, and theory of mind abilities to examine the unique roles and interplay of language and executive function in first-order and second-order false belief reasoning.

**Results:**

Results showed that language significantly uniquely predicted the children’s first-order as well as second-order false belief reasoning when controlling for the effects of age and executive function. Although executive function significantly predicted first-order FB reasoning when controlling for age, it was no longer a significant predictor of first-order FB reasoning when language was included in the model. However, executive function played a significant unique role in second-order FB reasoning when controlling for the effects of age and language.

**Discussion:**

The current findings suggest that language plays a greater role than executive function in Mandarin-speaking children’s theory of mind development and the contributors to theory of mind development vary in different levels of false belief reasoning.

## 1 Introduction

Preschoolers experience rapid development in theory of mind (ToM), the ability to understand one’s own or others’ mental states (e.g., desires, intentions, beliefs and feelings, etc.) and predict others’ behaviors according to their mental states (Premack and Woodruff, 1978). Four-year-olds begin to acquire first-order false belief (FB) reasoning (others have different beliefs from their own or reality), the hallmark ability of ToM (Wellman et al., 2001). During 5–6 years old, children begin to understand more advanced second-order FB reasoning (one person’s false beliefs about a third person’s false beliefs) (Perner and Wimmer, 1985). Regarding the mechanism of ToM development, researchers have proposed that there are multiple routes, including various aspects of language and executive function (EF) for children to develop ToM (Im-Bolter et al., 2016; Farrar et al., 2017). Substantial studies have documented that both language and EF play important roles in ToM development (Milligan et al., 2007; Devine and Hughes, 2014); however, most of previous research focused on the relations between first-order FB reasoning and language or EF, and did not concurrently examine the roles of language and EF in FB reasoning. To date, the effects of language and EF in higher-order ToM are far from clear, and the interplay of language and EF in ToM development remains inconclusive. To better understand how ToM develops as a function of intersections with other cognitive abilities, this study examined the unique roles and interplay of language and EF in Mandarin-speaking children’s first-order FB and second-order FB reasoning.

The crucial roles of various aspects of language in ToM development have been well established (Milligan et al., 2007). For instance, general language measured by tasks such as the Reynell Developmental Language Scales (Reynell and Huntley, 1985) and the Test of Early Language Development – 3 (Hresko et al., 1999) that assess children’s vocabulary, syntactic and semantic knowledge has been found playing an important role in ToM development (e.g., Cheung et al., 2004; Tardif et al., 2007). More specifically, sentential complement structure has been found playing a crucial role in first-order FB reasoning as well. According to linguistics determinism, sentential complement structure provides a representational format for FB reasoning (de Villiers, 2000). Different from other complex syntactic structures such as relative clauses, sentences with complement clauses, for example, *John thinks that Mary is at the office*, make it possible that the whole sentence is true, while the proposition is false. de Villiers (2000) and de Villiers and Pyers (2002) found that children’s earlier sentential complementation predicted their later first-order FB understanding, but not vice versa. Additionally, several training studies have attested to the importance of complementation in first-order FB reasoning (e.g., Lohmann and Tomasello, 2003; Mo et al., 2014).

In recent years, another aspect of language, namely verb factivity, has been found closely related to first-order FB as well as second-order FB reasoning. Verb factivity refers to a feature of predicates that presupposes speakers’ beliefs about the truth or falsity of propositions of complement clauses (Kiparsky and Kiparsky, 1971). For instance, *John knew/pretended/thought that the cake was in the box*, the factive verb *know* and the counter-factive verb *pretend* presuppose the speaker’s true and false belief about the location of the cake, respectively. By contrast, the non-factive verb *think* does not presuppose the speaker’s true or false belief about the location of the cake. Therefore, a comprehensive understanding of verb factivity requires monitoring others’ mental states. Specifically, the hearer needs to distinguish his own, the sentence subject’s, and the speaker’s beliefs from each other. It has been hypothesized that verb factivity conveyed by counter-factive verbs and FB reasoning are naturally related as both involve decoupling a false mental representation of reality (Chen et al., 2012). The findings from neuro-imaging research have indicated that verb factivity and first-order FB reasoning share neural basis (Cheung et al., 2012). Moreover, results from cross-sectional (Cheung et al., 2009; Li and Leung, 2020) as well as longitudinal (Kristen-Antonow et al., 2019; Siu and Cheung, 2022) studies have attested to a close link between verb factivity and first-order FB reasoning.

Apart from first-order FB reasoning, language plays an important role in second-order FB reasoning as well. For example, Hollebrandse et al. (2014) examined whether language supported the development of second-order FB reasoning by comparing 6- to 9-year-old Dutch-speaking children’s performance on a verbal and a low-verbal version of a second-order FB task. They found that the children significantly performed better in verbal than in low-verbal second-order FB tasks, which indicated that language might facilitate children’s explicit second-order FB understanding. Arslan et al. (2017) attested to a significant correlation between second-order syntactic recursion of relative clauses and second-order FB reasoning after the effects of age and simple working memory were removed. Kristen-Antonow et al. (2019) and Li and Leung (2020) found that verb factivity was closely related to second-order FB reasoning. Although a few studies have explored the relation between language and second-order FB reasoning, they were limited in assessing only one aspect of language, and not taking account of other potential cognitive contributors such as EF. Therefore, a clear picture of the role of language in second-order FB reasoning is yet to emerge.

Executive function (EF) encompasses a set of cognitive processes such as inhibitory control, cognitive flexibility, and working memory that underlie goal-directed behaviors (Diamond, 2013). Inhibitory control involves inhibiting responses to prepotent but irrelevant stimuli while pursuing a cognitively represented goal (Carlson and Moses, 2001). Cognitive flexibility refers to change perspectives based on a shift in rules or demands (Diamond, 2013). Working memory involves both holding and manipulating information in mind (Diamond, 2013). It has been proposed that EF is necessary for the emergence and expression of ToM ability (Carlson and Moses, 2001; Flynn et al., 2004; Benson et al., 2013; Devine and Hughes, 2014). To pass FB tasks, for example, the change-of-location FB tasks, in which participants are required to predict a story protagonist’s behavior based on his or her false belief about an object’s location (Wimmer and Perner, 1983), participants need to simultaneously hold in mind conflicting perspectives about an object’s location, to suppress their own prepotent knowledge of the object’s current location, and to predict story protagonist’s action according to their less salient false representation of the object’s location (Carlson and Moses, 2001). Numerous studies have found robust links between preschoolers’ first-order FB understanding and inhibition, cognitive flexibility and working memory independent of age and verbal ability (e.g., Hughes, 1998a; Henning et al., 2011; Devine and Hughes, 2014; Carlson et al., 2015; Duh et al., 2016).

To date, most of previous studies have focused on the relation between EF and first-order FB reasoning in preschoolers, the role of EF in older children’s higher-order FB reasoning has been relatively less examined. The literature on the relation between EF and advanced ToM has shown that EF is significantly correlated with advanced ToM in middle childhood (Charman et al., 2001), even when controlling for age and intelligence, or verbal ability (Perner et al., 2002; Devine et al., 2016; Wang et al., 2016). Moreover, findings from several longitudinal studies have indicated that early EF significantly predicts later advanced ToM (e.g., Hughes, 1998b; Austin et al., 2014; Lecce et al., 2017; Shahaeian et al., 2023). However, previous studies have produced inconsistent findings regarding which subcomponents of EF predict advanced ToM. For instance, Hughes (1998b) found that early inhibition significantly predicted later ToM, while Lecce et al. (2017) found that early working memory significantly predicted later ToM, and Austin et al. (2014) found that early attention shifting and working memory but not inhibition significantly predicted later ToM.

The findings from previous studies suggest that the relation between EF and ToM varies in children of different ages and in different levels of FB reasoning. For instance, the correlation between EF and first-order FB reasoning was significant for 3- and 4-year-olds but not for 2-year-olds (Müller et al., 2012). Inhibition rather than working memory significantly predicted 3- to 4-year-olds’ first-order FB performance (Carlson et al., 2002); while, working memory was significantly correlated with 4- to 6-year-olds’ (Perner et al., 2002) and predicted 4- to 8-year-olds’ (Arslan et al., 2017) second-order FB reasoning. Therefore, to better understand the relation between EF and ToM, it is necessary to examine the role of EF in different levels of FB reasoning throughout childhood.

Both language and EF play important roles in ToM development, and these two are closely related as well (Shokrkon and Nicoladis, 2022). For instance, success on the complements task requires one to hold different pieces of information in mind and inhibit a prepotent response to correctly answer a test question, suggesting that working memory and inhibitory control play important roles in language tasks (Tardif et al., 2007). To pass EF tasks such as the Dimensional Change Card Sort (DCCS) task (Zelazo, 2006), a certain level of language capacity is required to understand the *if*-*if*-*then* sentence structures. Therefore, EF and language should influence each other’s role in ToM development; however, only a few studies have examined the interplay of EF and language in ToM development.

Previous research on the interrelations among language, EF and ToM development have yielded mixed results. A few studies have found that EF fully mediates the role of language in FB reasoning. Low (2010) found that verbal ability was no longer a significant predictor of 3- and 4-year-olds’ first-order FB understanding after the effect of cognitive flexibility was removed. Similarly, in Farrant et al.’s (2012) longitudinal study, children’s earlier sentential complement ability failed to significantly predict their later FB understanding when controlling for cognitive flexibility. On the contrary, findings from other studies suggest that language may mediate the role of EF in FB reasoning. Jenkins and Astington (1996) found that 3- to 5-year-olds’ working memory no longer accounted for any unique variance in their first-order FB reasoning when controlling for language. In a study by Hughes (1998a), the strength of correlations between 3- and 4-year-olds’ EF (working memory and inhibition) and first-order FB understanding was no longer significant or reduced, after removing the effects of verbal and non-verbal abilities.

Previous studies on the relations among language, EF and FB reasoning have focused on preschoolers’ first-order FB reasoning, the interplay of language and EF in second-order FB reasoning beyond preschoolers is less studied and inconclusive. Moreover, to date, most research focuses on English-speaking children. Although previous studies on the role of language in ToM development in Mandarin-speaking children (e.g., Mo et al., 2014) have yielded similar results to those involving English-speaking children (e.g., Lohmann and Tomasello, 2003), and both Mandarin- and English-speaking children have displayed parallel trajectories in ToM development, the two groups differ in various aspects, such as EF development and the use of think-falsely verbs which are relevant to ToM development (Liu et al., 2008). For instance, Chinese preschoolers demonstrated advanced EF but not advanced ToM when compared with their U.S. counterparts (Sabbagh et al., 2006; Liu et al., 2008), indicating that Chinese preschoolers may not rely heavily on EF to develop their ToM. In Chinese, there are some specific verbs that express false beliefs such as *yiˇwéi* ‘falsely think’, and the use of such words in the test questions of FB tasks has been shown to improve Chinese-speaking children’s performance in FB understanding (Lee et al., 1999; Tardif et al., 2004). The daily use of those words or certain specific language structure such as complementation may provide children more opportunities to draw their attention to others’ minds, or provide them a scaffolding to represent others’ mental states. Children from diverse cultures may vary in the tendency to rely more on different factors in ToM development. To better understand the universality of the mechanisms underlying ToM development as well as specific experiential factors in ToM development, it is necessary to conduct research based on non-Western children speaking non-Indo-European languages and to investigate how language and EF work together in different levels of FB reasoning in children of a wider age range.

This study examined the roles of language and EF in Mandarin-speaking children’s first-order and second-order FB understanding. As various aspects of language and EF play crucial roles in ToM development, in this study, we examined three important aspects of language (verbal ability, sentential complement and verb factivity), and EF (cognitive flexibility, inhibitory control and working memory) in preschoolers and early primary schoolers to better capture the roles of language and EF in ToM development. Compared with first-order FB reasoning, the roles of language and EF in second-order FB reasoning are relatively under-explored. Therefore, our first goal was to examine whether the significant effects of language and EF on first-order FB extend to second-order FB reasoning or not. Based on previous meta-analytic studies on the relation between language or EF and first-order FB reasoning (Milligan et al., 2007; Devine and Hughes, 2014), and previous studies on the roles of language and EF in second-order FB or advanced ToM (e.g., Austin et al., 2014; Arslan et al., 2017), we expect to find that language and EF would play significant roles in both first-order FB and second-order FB reasoning. Since Chinese preschoolers demonstrate advanced EF but not advanced ToM when compared with their U.S. counterparts (Sabbagh et al., 2006; Liu et al., 2008), and in Chinese, there are think-falsely verbs which would facilitate ToM development, we also expect to find that language plays a greater role than EF in Mandarin-speaking children’s FB reasoning.

In addition, our second goal was to examine whether the roles of language and EF on second-order FB are greater than those on first-order FB reasoning, respectively. Since the stories in second-order FB tasks were more complex, for example, in sentence length and in the number of protagonists, they may be more demanding in language and EF capacity, we expect to find that language and EF play greater roles in second-order FB reasoning than in first-order FB reasoning.

Furthermore, since the interplay of language and EF in first-order FB reasoning remains unclear, and that in second-order FB reasoning is under-explored, our third goal was to examine whether language and EF influence each other’s role in first-order and second-order FB reasoning or not. Although previous studies on the interplay among language, EF and FB reasoning have yielded conflicting results, with some studies suggesting that language mediated the role of EF in FB reasoning (e.g., Jenkins and Astington, 1996), while some studies indicating the opposite (e.g., Farrant et al., 2012), we expect to find that language and EF partially mediate each other’s role in FB reasoning, since language and EF are closely related, and both have been found playing significant unique roles in ToM development (Milligan et al., 2007; Devine and Hughes, 2014; Arslan et al., 2017; Li and Leung, 2020). The current results will offer additional evidence on the roles and interplay of language and EF in first-order FB, especially in second-order FB reasoning from Mandarin-speaking children.

## 2 Materials and methods

This study is part of a large project on the language and cognitive development of Mandarin-speaking children. The participants, language and FB tasks in this study were included in previously published research (Li and Leung, 2020). Our current investigation focused on the unique, relative roles and interplay of language and EF in ToM development.

### 2.1 Participants

In this study, 160 native monolingual Mandarin-speaking children (age range: 50–90 months; mean age = 71 months; *SD* = 11 months, 82 boys) were randomly selected from a public kindergarten and from a public primary school in Shenzhen, a southern city in China. The kindergarten and primary school are in Nanshan and Longhua districts, of which the GDP ranked the first and the fourth out of 11 districts in Shenzhen city in 2022, respectively. We estimated that the social economic status of the children ranged from medium to high. According to teachers’ reports, they were free of language and cognitive deficits. Ethical approval for this study was provided by the Human Subjects Ethics Sub-committee at the Hong Kong Polytechnic University, and parent consent forms for the children were obtained before testing. The data from a child was dropped because he missed one first-order FB task. Therefore, subsequent analyses were based on the remaining 159 children (age range: 50–90 months; mean age = 71 months; *SD* = 11 months, 81 boys).

### 2.2 Materials

#### 2.2.1 Verbal ability measure

The Peabody Picture Vocabulary Test – Revised (PPVT-R) (Sang and Miao, 1990) was used to assess participants’ verbal ability. In each test trial, participants were required to select one from four objects or scenes in a picture according to the word they heard. The test was discontinued if the participant failed to correctly answer six trials among eight consecutive trials. There were 175 test trials, and each correct response was scored 1.

#### 2.2.2 Complementation measure

The memory for complements task (de Villiers and Pyers, 2002; Durrleman et al., 2016) was adapted to assess participants’ understanding of complementation. Twelve short stories were devised, each described a protagonist who made a mistake or told a lie or had a false belief. Each story was accompanied by two pictures and depicted by three sentences. For example, first, a picture was shown and a test sentence was played on a notebook computer (*Grandma says that there is an egg in the bowl*.), followed by another picture and a sentence (*But look, this is only a ball*.), and then a test question (*What did grandma say was in the bowl?*). Test sentences were constructed with the communication verb *shuō* ‘say’ as the main clause predicate. Each correct response was scored 1.

#### 2.2.3 Verb factivity measure

Participants’ comprehension of verb factivity was assessed by a truth value judgment task (Abbeduto and Rosenberg, 1985). After participants were seated, the experimenter told them that a girl hand puppet, *Xiǎohuā* (placed on the right side of participants) would tell them a short story accompanied by a picture (e.g., *This is Dà Péng. He sees a bottle on the cupboard. Dà Péng does not know that there is apple juice in the bottle*.), and then a teacher hand puppet (placed on the left side of participants) would ask them a test question (e.g., *So, is there any apple juice in the bottle?*). Three buttons, marked by labels of *shì* ‘yes’, *búshì* ‘no’ and *kěnéngba* ‘maybe’, respectively, were placed in front of the participants, and they were required to press one of them to make judgments.

Three verbs examined in the verb factivity test were reported here: *zhīdào* ‘know’ (a factive verb), *juédé* ‘think’ (a non-factive verb) and *jiǎzhuāng* ‘pretend’ (a counter-factive verb). These verbs were used as main clause predicates to construct test sentences with complements in three conditions: (1) affirmative main and complement clause predicates (“+ +” hereafter, e.g., *Mary knew that Paul was at home*.), (2) affirmative main clause predicate and negative complement clause predicate (“+ −” hereafter, e.g., *Mary knew that Paul was not at home*.) and (3) negative main clause predicate and affirmative complement clause predicate (“– +” hereafter, e.g., *Mary did not know that Paul was at home*.). Five test sentences with *zhīdào* ‘know’ and *juédé* ‘think’ were constructed in each of the three conditions, and with *jiǎzhuāng* ‘pretend’ were constructed only in “+ +” and “+ −” conditions, because *jiǎzhuāng* ‘pretend’ carries a sense of negation itself and is seldom used in negation. Therefore, there were 40 test sentences in total. From the perspective of verb factivity, correct responses to *zhīdào* ‘know’ in “+ +,” “+ −” and “– +” conditions are ‘yes’, ‘no’ and ‘yes’, respectively; to *juédé* ‘think’ in all three conditions are ‘maybe’ and to *jiǎzhuāng* ‘pretend’ in “+ +” and “+ −” conditions are ‘no’ and ‘yes’, respectively. Each correct response was scored 1. Test sentences were pseudo-randomized, with the same verb in the same condition occurring no more than two consecutive trials.

#### 2.2.4 Inhibition measure

The day-night stroop task (Gerstadt et al., 1994) was used to assess participants’ inhibitory control of the prepotent response of matching a word they say to an object shown. Participants were required to say *day*/*night* for each card showing the moon/the sun (Gerstadt et al., 1994). They received 16 test trials (eight sun cards and eight moon cards) in a fixed pseudo-random order with the same type of card occurring no more than two consecutive trials. No feedbacks were provided during the test. Each correct response was scored 1.

#### 2.2.5 Cognitive flexibility measure

The DCCS task was employed to assess participants’ cognitive flexibility, including a standard version (suitable for 2- to 5-year olds) and a border version (suitable for 5- to 7-year-olds) (Zelazo, 2006). Two target cards showing a red boat and a blue rabbit, respectively, were affixed to two bookends in front of the participants. Two sorting trays were placed with an approximately 30 cm-interval in front of the bookends. The standard version required participants to sort six cards (three red rabbit cards and three blue boat cards) according to one dimension (e.g., color) in the pre-switch phase, and then another six according to another dimension (e.g., shape) in the post-switch phase. The order of sorting dimensions in the two phases was counterbalanced. The experimenter repeated the rules before each test trial: “When playing the color/shape game, if the card is red/depicts a boat, then put it here; if the card is blue/depicts a rabbit, then put it here.” Participants received the post-switch trials if they passed the pre-switch phase by correctly sorting five or more out of the six pre-switch trials.

Participants received the border version if they passed the standard version by correctly sorting five or more out of the six post-switch trials. The border version included 12 test trials (three red rabbit cards and three blue boat cards with/without a black border), and required participants to sort cards with black border according to color, and those without black border according to shape. The experimenter repeated the rules before each test trial: “If there is a black border, then play the color sorting game; if there is no black border, then play the shape sorting game.” Participants had to correctly sort 9 or more out of the 12 trials to pass the border version. Test cards in both versions were in pseudo-random order, with the same card occurring no more than two consecutive trials. During the test, no feedbacks were provided. Participants’ performance was scored 0 if they failed the pre-switch phase, 1 if they passed the pre-switch phase, 2 if they passed the post-switch phase, and 3 if they passed the border version (Zelazo, 2006).

#### 2.2.6 Working memory measure

The backward digit span test (Davis and Pratt, 1995) was used to assess participants’ working memory. The test included 5 two-digit length practice trials, and 14 two- to eight-digit length test trials, with two trials for each length. Participants received test trials until they correctly responded to one practice trial, the test was discontinued if they failed all five practice trials. For test trials, a correct response to at least one of two digits with the same length led to the next two trials which were longer by one digit. The test was discontinued if participants failed to correctly respond to both trials with the same length. The span (the number of digits in the longest correct sequence, ranging from 0 to 7) was registered.

#### 2.2.7 False belief measures

Participants’ first-order FB understanding was assessed by two change-of-location tasks (Wimmer and Perner, 1983) and two unexpected-content tasks (Gopnik and Astington, 1988). In each change-of-location task, there were two protagonists (brother and sister; *Xiǎomíng* and mother). Brother/*Xiǎomíng* put a football/a cake in a box/bowl and then left the scene. Sister/Mother transferred the football/cake into a basket/box when brother/*Xiǎomíng* was absent. After a memory control question (*Where did brother/Xiǎomíng put the football/cake at the very beginning?*) and a reality control question (*Where is the football/cake now?*), participants were asked to predict where brother/son would look for the football/cake when they came back. In each unexpected-content task, participants were asked to guess what contents were in a M&M candy tube/cookie box, and then were shown the real contents after their responses. After a memory control question (*Did you remember what’s in the tube/box?*), participants were asked to predict their own and another protagonist’s beliefs about the contents of the tube/box before opening the tube/box. Each change-of-location task and unexpected-content task included one and two test questions, respectively. Each correct response was scored 1, and the maximum raw score of first-order FB tasks was 6.

Second-order FB understanding was assessed by four second-order FB tasks: the ice-cream van story (Perner and Wimmer, 1985), the soccer practice story (Miller, 2013a), the cake story (Miller, 2013b) and the hidden toy story (Astington et al., 2002). In each story, Protagonist A (*Chéngcheng*/*Xiǎotāo*/*Ziˇxuān*/*Dàwěi*) had a false belief about Protagonist B’s (*Fāngfang*/*Hàohao*/*Qíngqing*/*Lìli*) belief about the location of an object or event (ice-cream van/playing football/cake/airplane toy). At the end of each story, participants were asked to predict where Protagonist A (*Chéngcheng*/*Xiǎotāo*/*Ziˇxuān*/*Dàwěi*) would look for Protagonist B (*Fāngfang*/*Hàohao*) or the object mentioned (cake/airplane toy) before they correctly responded to all control questions. Each correct response was scored 1, and the maximum raw score for second-order FB tasks was 4.

Traditionally, participants receive a point when they pass both control and test questions in a FB task, and those who fail control questions of a FB task are excluded from data analysis, known as “Exclude System” as described by Sobel and Austerweil (2016) (e.g., Perner et al., 1987), or receive no score even they pass test questions, referred to as “Failure System” by Sobel and Austerweil (2016) (e.g., Astington and Jenkins, 1999). In this study, not all children passed the control questions for all four first-order or second-order FB tasks; some failed the control questions for one, two or three first-order or second-order FB tasks. If we excluded the participants who failed control questions for any one of the FB tasks, the sample size would be significantly reduced. Therefore, we did not exclude the children who failed control questions for FB tasks. If we assigned no scores to participants who failed control questions, we would be unable to distinguish them from those who passed control questions but failed test questions for FB tasks. For example, if child A and child B pass test questions for three out of four second-order FB task, but child A passes control questions for three while child B passes the control questions for four second-order FB tasks. In a traditional “Failure System,” both children would receive 3 for second-order FB tasks. Despite receiving the same score, we believe that they differ as least their ability to pass control questions for second-order FB tasks. Therefore, we multiplied the child’s raw score on first-order or second-order FB tasks by the proportion of the number of control questions-passed first-order or second-order FB tasks. For child A and child B mentioned above, their final second-order FB score will be calculated as 3*(3/4) = 2.25 and 3*(4/4) = 3, respectively. As a result, these two children were distinguishable based on their final scores on second-order FB tasks. Although the FB scores derived from the way used in this study were different from those derived from the conventional “Failure System” way, there were significant correlations between the scores for first-order FB and second-order FB tasks derived from the conventional “Failure System” way and those derived from the current way, respectively [First-order FB tasks: *r*(159) = 0.87, *p* < 0.001; second-order FB tasks: *r*(159) = 0.85, *p* < 0.001].

### 2.3 Procedure

Participants received the test individually in the respective kindergarten and primary school. The process of the testing was audio-recorded. Preschoolers received all tasks in three 30-min sessions. The first session included verbal ability and complementation tests; the second and third sessions included EF, FB, and factivity tests, with FB and factivity test trials equally distributed in the two sessions. Primary schoolers received all tasks in two 45-min sessions. The first session included verbal ability, complementation, and EF tests; the second session included FB and factivity tasks. First-order FB tasks were always administered before second-order FB tasks in each session. The order of FB and factivity tests was counterbalanced. Complementation and factivity test trials and FB questions could be played three times at the most if participants did not hear them clearly.

## 3 Results

In this section, we first report descriptive statistics of the data, and then results of simple correlation analyses, and those of path analyses which investigated the direct and indirect effects of language and EF on FB reasoning. The descriptive statistical and correlation analyses were conducted by using SPSS 26.0 software, and path analyses were conducted by using Amos 28.0 software.

### 3.1 Descriptive statistics

Table 1 shows means and standard deviations of each task. It demonstrates that the children performed at a high level on the cognitive flexibility, inhibition, complements and first-order FB tasks, but not on the verbal ability, verb factivity, working memory and second-order FB tasks. One-way ANOVA revealed that there were no gender differences in the performance on each task. Therefore, gender was not entered in any subsequent analyses.

**Table 1:** Means and standard deviations of each task (*N* = 159).

Variables (range)	Mean	*SD*
Verbal ability (0–175)	92.46	23.25
Complements (0–12)	9.52	3.88
Verb factivity (0–40)	22.85	7.20
Cognitive flexibility (0–3)	2.44	0.56
Inhibition (0–16)	15.14	2.22
Working memory (0–7)	1.87	0.98
First-order FB (0–6)	4.56	1.76
Second-order FB (0–4)	1.32	1.30

### 3.2 Correlations

Table 2 demonstrates correlations among variables tested. It shows that age was significantly correlated with all the other variables, and all subcomponents of language and EF were significantly correlated with first-order FB and second-order FB reasoning. Consistent with previous studies (Shokrkon and Nicoladis, 2022), language was significantly correlated with EF as well. Verbal ability was significantly correlated with cognitive flexibility and working memory, complements was significantly correlated with inhibition and working memory, and verb factivity was significantly correlated with all three subcomponents of EF. Overall, the correlation analyses indicate that language, EF and FB abilities were significantly intercorrelated, providing us preliminary bases to examine the unique roles and interplay of language and EF in first-order FB and second-order FB reasoning.

**Table 2:** Correlations of variables tested (*N* = 159).

Variables	1	2	3	4	5	6	7	8
1. Age	–	0.51^**^	0.38^**^	0.52^**^	0.44^**^	0.28^**^	0.38^**^	0.42^**^
2. Verbal ability		–	0.30^**^	0.39^**^	0.37^**^	0.15	0.34^**^	0.38^**^
3. Complements			–	0.37^**^	0.16	0.22^**^	0.31^**^	0.46^**^
4. Verb factivity				–	0.34^**^	0.23^**^	0.28^**^	0.38^**^
5. Cognitive flexibility					–	0.09	0.28^**^	0.20^*^
6. Inhibition						–	0.19^*^	0.20^**^
7. Working memory							–	0.37^**^
8. First-order FB								–
9. Second-order FB	0.36^**^	0.36^**^	0.31^**^	0.39^**^	0.32^**^	0.17^*^	0.35^**^	0.47^**^

^*^p < 0.05, ^**^p < 0.01 (two-tailed).

### 3.3 Path analyses

We conducted two path analyses to examine the roles of language and EF in first-order FB and second-order FB reasoning. In each analysis, first-order FB and second-order FB were the dependent variables, language or EF was the independent variable or the mediator according to specific purpose. We performed path analysis by using the direct and indirect effects based on bootstrap procedures (1,000 samples) and bias-corrected bootstrap confidence interval (95%). We computed factor scores for language and EF based on all three aspects of language and EF, respectively, and used them in the analyses. Table 3 presents the factor loadings and communalities for the language and EF items.

**Table 3:** Factor loadings and communalities for language and EF items (*N* = 159).

Latent variable	Indicator	Factor loading	Communality
Language	Verb factivity	0.79	0.62
	Verbal ability	0.74	0.55
	Complements	0.73	0.53
EF	Working memory	0.78	0.49
	Cognitive flexibility	0.70	0.29
	Inhibition	0.54	0.61

**Figure 1:**
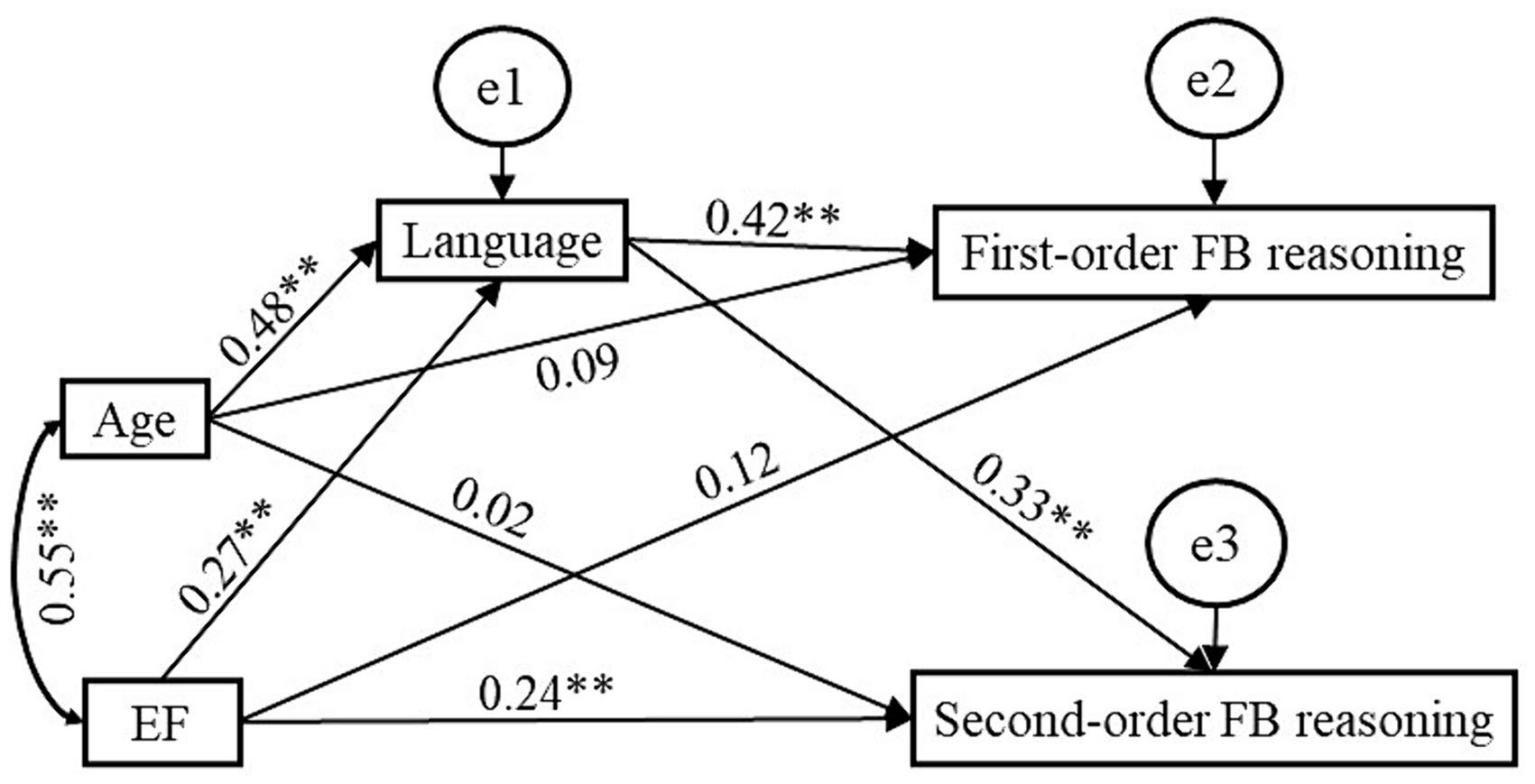
Path diagram for the indirect effect of language on the EF-first-order FB reasoning and EF-second-order FB relations. ^*^p < 0.05, ^**^p < 0.01.

**Figure 2:**
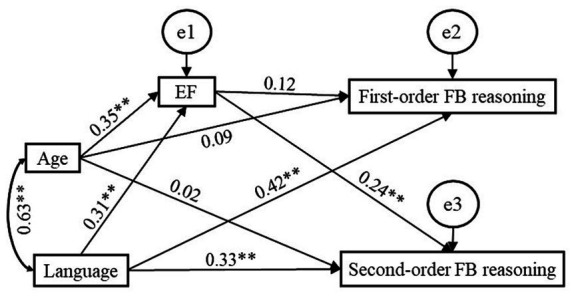
Path diagram for the indirect effect of EF on the language-first-order FB reasoning and language-second-order FB relations. ^*^p < 0.05, ^**^p < 0.01.

Figures 1, 2 illustrate the effects of language and EF on first-order FB and second-order FB reasoning. The results reveal that language played a significant unique role in both first-order and second-order FB reasoning, and the effect of language on first-order FB reasoning (*β* = 0.42, *p* = 0.002) was higher than that on second-order FB reasoning (*β* = 0.33, *p* = 0.001); whereas, EF played a significant unique role in second-order FB reasoning, but not in first-order FB reasoning. For second-order FB reasoning, language (*β* = 0.33, *p* = 0.001) played a greater role than EF (*β* = 0.24, *p* = 0.002) in it.

**Figure 3:**
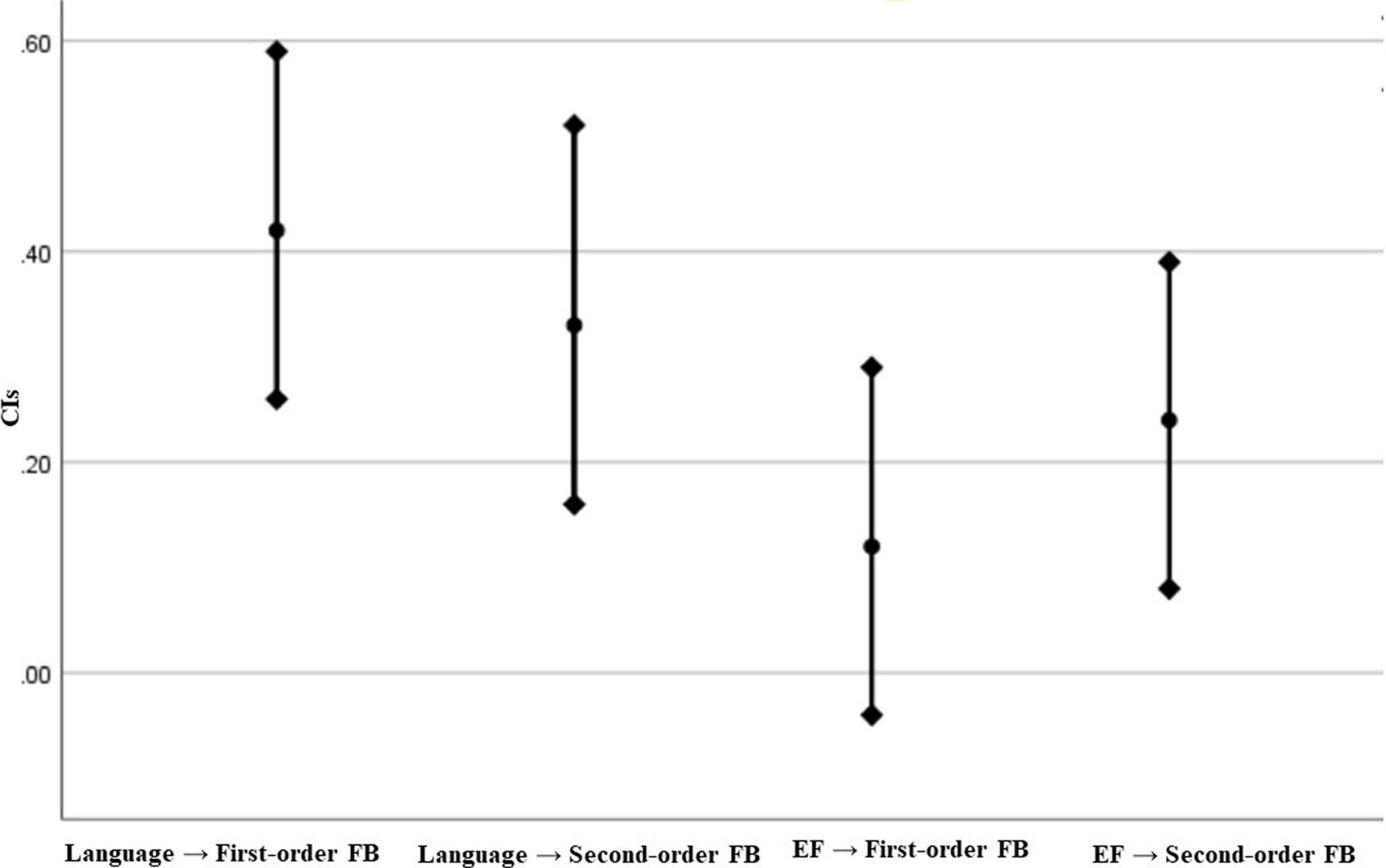
Difference between standardized beta coefficients.

To test whether the effects of language on first-order FB (*β* = 0.42, CI_95%_ = [0.26; 0.59]) and second-order FB reasoning (*β* = 0.33, CI_95%_ = [0.16; 0.52]), the effects of EF on first-order FB (*β* = 0.12, CI_95%_ = [−0.04; 0.29]) and second-order FB reasoning (*β* = 0.24, CI_95%_ = [0.08; 0.39]), the effects of language (*β* = 0.42) and EF (*β* = 0.12) on first-order FB reasoning, and the effects of language (*β* = 0.33) and EF on second-order FB reasoning (*β* = 0.24) were significantly different from each other, we followed the method suggested by Cumming (2009) in which significant differences exist between beta coefficients when the corresponding 95% confidence intervals overlap by less than 50% of the length of one confidence interval arm. For each comparison, we calculated half of the average of the overlapping confidence intervals and added it to the beta weight lower bound estimate. It showed that the appropriate confidence intervals overlapped by more than 50% (see Figure 3), indicating that there were no significant differences between the effects of language on first-order FB and on second-order FB reasoning, or between the effects of EF on first-order FB and on second-order FB reasoning, or between the effects of language and EF on first-order FB reasoning, or between the effects of language and EF on second-order FB reasoning.

We summarized the direct and indirect effects of language and EF in Table 4. It reveals a significant indirect effect of EF on first-order FB reasoning (*β* = 0.11, *p* = 0.002), and a significant total effect of EF on first-order FB (*β* = 0.23, *p* = 0.01). With the inclusion of the mediator, the effect of EF on first-order FB reasoning was insignificant (*β* = 0.12, *p* = 0.11). This shows that language significantly fully mediated the relationship between EF and first-order FB reasoning. In contrast, the indirect effect of language on first-order was insignificant (*β* = 0.04, *p* = 0.09), and the total effect of language on first-order FB reasoning was significant (*β* = 0.42, *p* = 0.002), and the direct effect of language on first-order FB reasoning was still significant (*β* = 0.46, *p* = 0.002) when the mediator was included. This shows that EF did not significantly mediate the role of language in first-order FB reasoning. For second-order FB reasoning, the total, direct and indirect effects of EF and language on it were significant, which indicates that EF and language significantly partially mediated each other’s role in second-order FB reasoning.

**Table 4:** Summary of the results of path analyses (*N* = 159).

Effect Path	Standardized effect coefficients		
	Total	Direct	Indirect
EF → Language → First-order FB	0.23^*^	0.12	0.11^**^
Language → EF → First-order FB	0.46^**^	0.42^**^	0.04
EF → Language → Second-order FB	0.33^**^	0.24^**^	0.05^*^
Language → EF → Second-order FB	0.41^**^	0.33^**^	0.09^**^

^*^p < 0.05, ^**^p < 0.01 (two-tailed).

## 4 Discussion

The current findings partially support our hypotheses. We found that language significantly uniquely predicted Mandarin-speaking children’s first-order and second-order FB reasoning when controlling for the effects of age and EF. EF played a significant unique role in second-order FB reasoning, but not in first-order FB reasoning. Regarding the interplay of language and EF in FB reasoning, language significantly fully mediated the effect of EF on first-order FB reasoning, and language and EF significantly partially mediated each other’s role in second-order FB reasoning.

### 4.1 Language and false belief reasoning

Our results are consistent with previous studies in that language played a significant unique role in first-order FB reasoning (e.g., de Villiers and Pyers, 2002; Cheung et al., 2009; Li and Leung, 2020), even when controlling for the effect of EF (e.g., Tardif et al., 2007). In addition, the current finding offers more evidence to the facilitating role of language in second-order FB reasoning (e.g., Hollebrandse et al., 2014; Arslan et al., 2017). Since the stories in second-order FB tasks were more complex than those in first-order FB tasks, we initially anticipated that language would play a greater role in second-order FB reasoning than in first-order FB reasoning. However, contrary to our expectation, we found that language played a greater role in first-order FB reasoning than in second-order FB reasoning. This finding suggests that the children in the current sample leaned more heavily on language to understand first-order FB reasoning than to understand second-order FB reasoning. The crucial role of language in first-order FB development has been well established in existing literature. Longitudinal and training studies have indicated that language plays a pivotal role in aiding children to build representation of other’s mental states (e.g., de Villiers and Pyers, 2002; Lockl and Schneider, 2007; Mo et al., 2014). Compared with first-order FB reasoning, second-order FB tasks are more complex in stories involved, and the primary distinction between first-order FB reasoning and second-order FB reasoning might lie in the information-processing requirements of the tasks. Lockl and Schneider (2007) proposed that once children have acquired basic language abilities that help them to develop first-order FB reasoning, their performance on second-order belief reasoning might largely depend on their comprehension of the complex stories used in second-order FB tasks. According to Lockl and Schneider’s (2007) proposal, the nature of the role of language in first-order FB reasoning may differ from that in second-order FB reasoning. Specifically, language supports the emergence of first-order FB reasoning, and the expression of second-order FB reasoning (Lockl and Schneider, 2007; Polyanskaya et al., 2022). Nevertheless, to date, the role of language in the transition from first-order FB to second-order FB reasoning remains unclear, and further research is needed to investigate this issue in more detail.

### 4.2 Executive function and false belief reasoning

Out of our expectation, EF did not play a significant unique role in first-order FB reasoning in this study. This finding is inconsistent with those from previous studies which found a significant predicting role of EF in first-order FB reasoning (e.g., Henning et al., 2011; Duh et al., 2016). The use of different controlled variables in path analyses may be one of the explanations for the discrepancies. In this study, we assessed three important aspects of language: verbal ability, syntactic complements and semantic verb factivity, and all three aspects of language were controlled in the path analyses predicting the role of EF in FB reasoning. By contrast, in previous studies, the controlled variables only included one aspect of language such as verbal ability (Carlson et al., 2015; Lecce et al., 2017) or syntactic competence (Henning et al., 2011), or no language competence (Duh et al., 2016). When controlling for age only, but not for language, we also found that EF significantly predicted first-order FB reasoning, which is in line with previous studies (e.g., Austin et al., 2014; Duh et al., 2016).

Another possible explanation for the discrepancies may be attributed to the variations in the age of the children across this and previous studies. The children in this study were older (mean age: 71 months) than those in previous studies (mean age: 48 to 59 months) (e.g., Henning et al., 2011; Carlson et al., 2015). It is noteworthy that we did not included children younger than 4 years old. Given that findings from previous studies have demonstrated diverse associations between EF and first-order FB reasoning among children of different age groups (Müller et al., 2012), the inclusion of younger children comparable to those in previous studies may yield a different picture of the relation between EF and first-order FB reasoning.

Although EF did not play a significant unique role in Mandarin-speaking children’s first-order, it significantly predicted their second-order FB reasoning after the effects of age and language were removed. Our findings indicate that EF played a greater effect on Mandarin-speaking children’s second-order FB reasoning than on their first-order FB reasoning, which lends support to Devine and Hughes’s (2014) proposal that the correlation strength between EF and FB tasks is contingent upon on the EF load required by the tasks. Specially, FB tasks demanding higher levels of EF are anticipated to exhibit stronger correlation with EF. In this study, second-order FB stories were more complex than first-order FB stories in the number and length of sentences, and in the number of protagonists, thus requiring participants to take more effect to retain and manipulate the story details, to switch flexibly among different protagonists’ mental states, and to suppress their own or others’ false beliefs. Therefore, the current second-order FB tasks impose more EF demands than first-order FB tasks, leading to an expectedly stronger correlation with EF.

### 4.3 Language, executive function and false belief reasoning

Regarding the relations among language, EF and FB reasoning, we did not find a mediation role of EF on the relation between language and first-order FB reasoning as previous studies (Low, 2010; Farrant et al., 2012). Instead, the role of EF in first-order FB reasoning was completely mediated by language in this study, which is in line with the findings from Jenkins and Astington (1996) and Hughes (1998b). However, for second-order FB reasoning, as expected, language and EF partially mediated each other’s role in it, and the effect of language was greater than that of EF. Together, we found that language played a greater role than EF in 4- to 7-year-old Mandarin-speaking children’s first-order and second-order FB reasoning.

The current findings that language played a greater role than EF in Mandarin-speaking children’s first-order and second-order FB reasoning support our hypothesis. Chinese children exhibit higher levels of EF than their U.S. counterparts (Sabbagh et al., 2006); however, their ToM is comparable to their U.S. counterparts’ (Liu et al., 2008). The findings of Sabbagh et al. (2006) and Liu et al. (2008), together suggest that Chinese children may not heavily rely on EF to develop their ToM. The current findings indicate that Chinese children relied more on language than on EF in FB reasoning. A possible explanation may be that in Chinese, there are some specific verbs that express false beliefs such as *yiˇwéi* ‘falsely think,’ and the use of those words in everyday conversations directly exposes children to instances of false beliefs, enabling them to observe others’ mental states and understand diverse perspectives on an event. This exposure would play a facilitating role in developing their ToM ability. However, the underlying reasons for why Chinese children tend to rely more on language to develop ToM warrant further cross-cultural research.

While this study focused on typically developing children, our findings align with research involving deaf and hard-hearing (DHH) children in that language plays an important role in FB reasoning. The literature on DHH children has shown that language is a key factor in their ToM development (e.g., Schick et al., 2007). Specifically, DHH children born to hearing parents often experience delayed language exposure, which correlates with their delays in ToM development (e.g., Stanzione and Schick, 2014; Walker et al., 2017). In contrast, DHH children who receive cochlear implants early or have access to signed language from a young age, have greater access to language and conversational experiences (Dettman et al., 2007), and develop ToM comparable to their hearing peers (e.g., Schick et al., 2007; Sundqvist et al., 2014; Yu et al., 2021). Beyond ToM, language also plays an important role in the EF development of DHH children (e.g., Jones et al., 2020; Goodwin et al., 2022). Studies have demonstrated that language mediates the EF differences between DHH and hearing children (e.g., Botting et al., 2017; Merchán et al., 2022). Despite this, few studies, to our best knowledge to date, have thoroughly explored the interplay between language and EF in the ToM development in DHH children. Therefore, the extent to which language influences the role of EF in ToM development in DHH children or the reverse remains an open question.

Our findings suggest that language plays a greater role than EF in FB reasoning in monolingual children, but the scenario may be different for bilingual children. Previous studies have shown that bilingual children often score lower on language tests than their monolingual counterparts (e.g., Carlson and Meltzoff, 2008; Bialystok and Viswanathan, 2009; Diaz and Farrar, 2018). However, they demonstrate an advantage in ToM development over their monolingual peers (e.g., Goetz, 2003; Farhadian et al., 2010; Schroeder, 2018), and this bilingual advantage in ToM may be explained by a bilingual advantage in EF (e.g., Bialystok and Senman, 2004; Kovács, 2009; Buac and Kaushanskaya, 2020). Therefore, the relative roles of language and EF in FB reasoning may vary between bilingual and monolingual children.

### 4.4 Limitations

In spite of the contributions to the literature on the relations among language, EF and ToM development, this study has several limitations. The first is that the day-night stroop task is too simple for the children in this study, as their performance on this task approached to the ceiling. In further research, more appropriate tasks should be employed to assess participants’ EF ability. The second limitation is that the use of verbal EF and FB tasks. Although we controlled for the effect of language when examining the role of EF in FB reasoning, the use of low-verbal or non-verbal EF and FB tasks would enable us to better elucidate the relation between EF and FB reasoning. The third is that the participants’ age range is limited (from ages 4 to 7), thus the current conclusions may not generalize to the roles of EF and language in ToM development before or beyond this age range. In this study, even the older children performed poorly on verb factivity, working memory and second-order FB tasks. The inclusion of children with a wider age range may contribute to a better investigation on the roles of language and EF in higher-order FB reasoning in further research. In addition, social environmental factors such as the number of siblings, economic social status and parents’ education background that may contribute to the relations among language, EF and ToM development were not available in this study. The current sample was collected from a kindergarten and a primary school in Shenzhen city. Due to the limited scope of this sample, its representativeness may be insufficient, thereby limiting the generalizability of the current findings to other samples characterized by distinct social environmental factors.

In sum, this study added to our understanding of the roles of language and EF and how they work together in ToM development. The current findings suggest that language plays a more prominent role than EF in 4- to 7-year-old Mandarin-speaking children’s first-order and second-order FB reasoning. In this study, we only examined the roles of language and EF in ToM development. In the course of children’s language, EF and ToM development, ToM could also play a predictive role in language and EF development. For further research, training or longitudinal studies would allow stronger inferences to the roles and interactions of language and EF in ToM development, and the directionality of effects among language, EF and ToM.

## Data availability statement

The raw data supporting the conclusions of this article will be made available by the authors, without undue reservation.

## Ethics statement

The studies involving humans were approved by the Human Subjects Ethics Sub-committee at the Hong Kong Polytechnic University (reference number: HSEARS20161103002). The studies were conducted in accordance with the local legislation and institutional requirements. Written informed consent for participation in this study was provided by the participants’ legal guardians/next of kin. Written informed consent was obtained from the individual(s), and minor(s)’ legal guardian/next of kin, for the publication of any potentially identifiable images or data included in this article.

## Author contributions

HL: Writing – original draft, Methodology, Investigation, Funding acquisition, Data curation, Conceptualization. M-TL: Writing – review & editing, Supervision, Conceptualization.
